# Associations of Prenatal Socioeconomic Status and Childhood Working Memory: A Structural Equation Modeling Approach

**DOI:** 10.3390/children12050537

**Published:** 2025-04-23

**Authors:** Shelley H. Liu, David Bellinger, Kristen Dams-O’Connor, Jeanne A. Teresi, Ivan Pantic, Sandra Martínez-Medina, John Chelonis, Martha M. Téllez-Rojo, Robert O. Wright

**Affiliations:** 1 Department of Population Health Science and Policy, Icahn School of Medicine at Mount Sinai, New York, NY 10029, USA; 2 Department of Neurology, Boston Children’s Hospital, Boston, MA 02115, USA; 3 Department of Rehabilitation and Human Performance, Icahn School of Medicine at Mount Sinai, New York, NY 10029, USA; 4 Department of Neurology, Icahn School of Medicine at Mount Sinai, New York, NY 10029, USA; 5 Stroud Center, Columbia University Department of Medicine, New York State Psychiatric Institute, New York, NY 10032, USA; jat61@cumc.columbia.edu; 6 Department of Environmental Health Sciences, Columbia University, New York, NY 10038, USA; 7 National Institute of Perinatology, Mexico City 11000, Mexico; sandra.martinez@inper.gob.mx; 8 Division of Neurotoxicology, National Center for Toxicological Research, U.S. Food and Drug Administration, Jefferson, AR 72079, USA; 9 Center for Nutrition and Health Research, National Institute of Public Health, Cuernavaca 62100, Mexico; mmtellez@insp.mx; 10 Department of Environmental Medicine, Icahn School of Medicine at Mount Sinai, New York, NY 10029, USA

**Keywords:** socioeconomic status, working memory, prenatal exposures, structural equation modeling

## Abstract

Objective: To determine if prenatal socioeconomic status (SES) is associated with childhood working memory (WM), we constructed a more precise, integrative measure of WM using variables from multiple tasks that may provide a more representative measure of WM. Study Design: We used data from a prospective birth cohort study in Mexico City, Mexico, with N = 515 children aged 6–9 years. Prenatal SES was measured using the Mexican Association of Marketing Research and Public Opinion Agencies (AMAI) index. We created a latent variable for nonverbal working memory using multiple tasks (Cambridge Neuropsychological Test Automated Battery spatial working memory, operant chamber Delayed Match to Sample and Incremental Repeated Acquisition). Structural equation models were used to assess associations between prenatal SES and nonverbal working memory, adjusting for child demographics (e.g., age and sex), prenatal exposures (e.g., exposures to lead, arsenic, and secondhand smoke), and family (current SES, maternal IQ) variables. Results: Children had a mean age of 6.6 years [SD 0.6], and 50.5% were boys. Using confirmatory factor analysis, we constructed a latent variable of nonverbal working memory, which was measurement invariant across child sex. Prenatal SES was associated with childhood nonverbal working memory (standardized factor loading = 0.17; *p* = 0.004). These associations were modified by child sex. Higher prenatal SES was significantly associated with higher childhood WM in females (standardized factor loading = 0.26; *p* = 0.002), but not in males. Conclusions: Prenatal socioeconomic status is a predictor of childhood working memory, but it may be a stronger predictor for girls compared with for boys.

## 1. Introduction

During early life, rapid and sequential changes in brain development are especially sensitive to “insults”, which can include environmental influences such as socioeconomic status (SES). Prenatal SES is associated with neonatal brain morphology [1], and socioeconomic disadvantage during pregnancy is associated with neurological abnormalities and autonomic nervous system dysfunction during childhood [2]. Thus, the detrimental effects of early socioeconomic disadvantage on neurodevelopment may persist across the lifespan [3]. However, most of the research that has examined the effects of SES on childhood executive functions (EFs) has examined SES post pregnancy. Executive functions (EFs) are high-level supervisory cognitive skills vital for a child’s social and academic success [4,5,6,7,8,9,10,11,12]. Studies of SES and childhood EF have yielded mixed findings, with a recent meta-analysis providing evidence of small to moderate effects [13].

Another possibility for these mixed findings is that conceptualizations of SES vary across studies and cultures. In the United States, SES is often operationalized using income or education, or less frequently a combination of the two, while in Europe, it is more commonly operationalized using occupation. However, these single-item indicators do not capture the multi-factorial nature of SES. While income is often used as a proxy for SES, wealth has been suggested to be even more important for health, as wealth can buffer the effects of temporary low income, and wealth has been shown to vary substantially across individuals with similar income levels [14]. Wealth has been measured using questions such as car or home ownership or information on liquid assets. In Mexico, SES is often measured using a validated, multi-item weighted index [15] that encompasses not only the educational level of the head of household, but also living situation and wealth (having a computer, appliances such as water heaters, washing machines, gas/electric stoves, soil/cement floors; the number of rooms/bathrooms, cars, etc.). This culturally sensitive index may provide a more holistic measure of SES for our cohort based in Mexico. Further, although SES is a construct that changes over time, most studies conceptualize it as a stable construct measured at study baseline and then used as a predictor [16]. Childhood SES has been shown to impact later health, independent of adult SES, and using SES measured at a single life stage may be inadequate to determine the full impact of SES on health [14].

Adding to the complexity of studying SES effects on EFs is that because EFs are high-level skills, they can be challenging to measure precisely. Often, an EF component is measured using a single performance task. For example, working memory (WM), a core component of EF that refers to sustaining one’s focus and keeping information in mind, is a broad construct that has been operationalized differently across studies, although there are some shared similarities in procedures that assess this construct. WM is often measured using a single task, which requires the child to remember information and update that memory as the task progresses. For example, WM may be assessed via a delayed matching-to-sample task, in which the child must remember what stimulus appeared at the beginning of a trial and then make the appropriate response, with the possibility that the stimulus to remember will be different for each trial.

However, research in the neurodevelopment and psychometrics literature has demonstrated that while using a single task to tap into an EF component can yield important information, single tasks can also be noisy indicators of the EF component [17]. This is because a child’s performance on a given cognitive task can also depend on non-EF cognitive processes; this idea is known as task impurity [18]. Moreover, there can be task-specific variance, in which there is error due to the specific task and not due to the underlying EF component being measured [18].

To address this, latent variable approaches [7,8,19,20,21] have been used to improve the precision of modeling an EF component. Rather than using a single task, a combination of tasks is used to tap into a single EF component, allowing researchers to extract what is common among the tasks to provide a more precise measure of the EF component. Further, because cognitive domains are inherently complex and multi-determined, a combination of tasks yields a more comprehensive picture of the underlying construct with greater measurement precision across the full range of functions. However, latent variable approaches remain underused when studying the effects of risk factors on EFs, which tends to rely on single-task analyses [19,22,23,24,25,26,27,28,29,30,31].

Here, our goal is to study whether prenatal SES is associated with childhood WM. The importance of childhood working memory on pediatric health is multifold: Childhood working memory is associated with academic performance [32,33,34,35,36,37,38,39,40,41,42] and health outcomes such as overweight and obesity [43,44,45,46,47,48,49,50,51] in later life. Working memory is important for the development of reading [34,38], arithmetic skills [35,36], mathematics [39], and other educational achievement skills [37,40]. Poorer childhood working memory is found to be associated with higher dropout risk in high school [41], and worse academic and occupational functioning in late adolescence and young adulthood [42]. Deficits in childhood working memory are related to neurodevelopmental disorders [45] such as attention deficit hyperactivity disorder [46,47], dyslexia [48], dyscalculia [49,50], and major depression disorder [51]. In this analysis, we constructed a more precise, integrative measure of WM using measures from multiple tasks that may provide a more representative measure of WM to study risk factors that impact childhood WM.

## 2. Methods

### 2.1. Data

Mother–child dyads included in the present study were recruited during the second trimester of pregnancy, as part of the Programming Research in Obesity, Growth, Environment and Social Stressors (PROGRESS) cohort. Participants were recruited through the Mexican Social Security System in Mexico City, Mexico. Written informed consent was obtained from all participants, and study protocols were approved by institutional review boards at the Brigham and Women’s Hospital, Icahn School of Medicine at Mount Sinai, and the Mexican National Institute of Public Health. The PROGRESS cohort initially comprised N = 948 children born to pregnant mothers recruited to the cohort. Of the original cohort, N = 406 children did not have scores for all three WM tasks at age 6. We also excluded the following numbers of participants who were missing covariate information: An additional N = 16 mother–child dyads were missing information regarding the mother’s IQ, and an additional N = 11 mother–child dyads were missing current SES information. Thus, the analytic sample for the present study was N = 515 mother–child dyads, who had complete data on prenatal SES, WM tasks, and covariates. Anonymized data were accessed on 10 April 2021.

### 2.2. Measures

Prenatal socioeconomic status: Prenatal SES was measured using a validated approach developed by the Mexican Association of Marketing Research and Public Opinion Agencies (AMAI) [15], using a weighted sum of thirteen culturally relevant indicators (e.g., the education level of the head of household, type of flooring, automobiles, etc.) to define 6 levels of SES, known as the 13 × 6 rule.

### 2.3. Current SES

Current SES was measured using an updated multi-item index from the AMAI, using 8 indicators to define 7 levels of SES, known as the 8 × 7 rule.

### 2.4. Working Memory Tasks

To develop a measurement model for WM, we used three nonverbal tasks, which were the CANTAB spatial working memory, and two behavioral tasks from the National Center for Toxicological Research-Operant Test Battery (NCTR-OTB): delayed matching-to-sample (DMTS) and incremental repeated acquisition (IRA). The selection of task variables for inclusion was based on theory-driven considerations [52] of the optimal indicators that represent different facets of the task. Following best practices [53], we included three pairs of non-correlated indicators for WM to ensure the identifiability of the model.

### 2.5. Cambridge Neuropsychological Test Automated Battery (CANTAB) Spatial Working Memory (CSWM) [54,55,56]

The participant was tasked with searching for hidden tokens within boxes, with increasing difficulty depending on the number of boxes. If a token was found inside a particular box, it would not be found again in that box during that trial, thus necessitating the participant to remember where the tokens had been located. We extracted two indicators: between search errors, which refer to when a participant returns to search a box where a token had already been located; and strategy, with higher scores indicating inefficient strategy.

### 2.6. Delayed Matching to Sample (DMTS)

The DMTS task has been described in detail elsewhere [57]. Briefly, the participant observed a shape that was presented on the center of three press plates. After the subject pressed the shape, the light was extinguished, and the subject had to choose the press plate with the matching shape from the three press plates illuminated with different shapes. Random delays of 1, 2, 4, 8, 16, and 32 s were used. The task lasted for 15 min, or until the participant had correctly completed 60 trials (and earned 60 tokens). From this task, we extracted two variables: overall accuracy, which is the number of correct choices divided by the total number of choices times 100, and overall correct choice latency time, which was the average time in seconds to press the correct choice. The overall correct choice latency time was chosen, rather than overall choice latency, as we conceptualize the time that it takes a child to make a correct response to be related to WM, with short correct choice latency times reflecting greater confidence in the answer. Furthermore, the exclusion of incorrect choice latencies likely reduces the contribution of attention in this model since children that tend to be “off task” will likely not respond as quickly and will more likely make an incorrect choice.

### 2.7. Incremental Repeated Acquisition (IRA)

The IRA tasks a participant with learning increasingly longer sequences of lever presses, with the goal to collect as many tokens as possible [58]. Briefly, one of six lights was illuminated, and the subject had to learn which of four response levers to press when that light was on to obtain a token. After the subject earned 3 tokens, a second light was illuminated, and the subject had to figure out which of the 4 response levers to press to turn off that light. Once that light was turned off, the first light was turned on, and the subject had to remember which lever to press to earn a token. This task continued, increasing the number of levers in the sequence until the subject successfully completed 3 six-lever sequences without making any errors or until 15 min had elapsed. We extracted two indicators for this task: memory accuracy, which was the total number of correct lever presses after the first time a particular stimulus light was illuminated (i.e., when the subject should have known which lever to press without searching) divided by the total number of lever presses after the first time that stimulus light was illuminated, and percent of task complete, which was the total number of errorless sequences completed divided by the total possible (18; 3 at 6 levels).

### 2.8. Covariates

We adjusted for maternal IQ, child sex, child age, and current SES. In secondary analyses, we additionally adjusted for prenatal exposure to neurotoxicants—lead, arsenic, and secondhand smoke exposure. We used maternal blood lead and arsenic concentrations measured at the second trimester of pregnancy, and self-reported exposure to secondhand smoke indoors during the second trimester of pregnancy.

### 2.9. Developing a Measurement Model for Childhood WM

We used confirmatory factor analysis (CFA) to test a one-factor model for WM using the six indicators from three tasks, allowing for correlation between indicators of the same task. We assessed the goodness of fit using a chi-squared test, which compared the covariance matrix of the observed variables to the covariance matrix predicted by the model, with a good fit indicated if the *p*-value for the chi-squared test was non-significant (*p* > 0.05). We also assessed the root mean squared error of approximation (RMSEA), with good fit indicated for RMSEA < 0.05. Further, we report the Comparative Fit Index (CFI), with good fit indicated for CFI > 0.95, and the standardized root mean square residuals (SRMR), with good fit indicated for SRMR < 0.08 [59,60].

Finally, we tested for measurement invariance by child sex [28], using increasingly stringent thresholds to test for strong invariance (factor loadings and intercepts are the same for boys and girls). We used a series of chi-squared tests to compare the model fit for configural invariance, weak invariance, and strong invariance, with non-significant results indicating measurement invariance by sex.

### 2.10. Associations Between Prenatal SES and WM

We used a structural equation model to assess associations between prenatal SES and WM, adjusted for covariates. To test whether the association of prenatal SES and WM differed by child sex, we used multigroup SEMs, with child sex as the grouping variable. We fit two models—a free model, in which the effect of prenatal SES is allowed to differ by child sex (with all other paths constrained to be the same for both boys and girls), and a constrained model in which the effect of prenatal SES is constrained to be the same for boys and girls (with all other paths constrained to be the same for both boys and girls). We then used a chi-squared test to assess whether the effect of prenatal SES differs significantly by child sex, with a cutoff of significance at alpha = 0.05. As neurodevelopment is sexually dimorphic, we also reported sex-stratified results, in which we used a multigroup SEM, with child sex as the grouping variable, and constrained factor loadings and correlations among the WM indicators to be the same across child sex. We allowed the paths of the predictors (prenatal SES and covariates) on WM to vary by child sex.

For missing data, we did not use imputation and did not use full information maximum likelihood for the structural equation modeling, as we did not know the underlying mechanism for the missing data; hence, we chose to use complete data here as we had a large sample size.

Analyses were conducted in R (version 4.5.0) using the lavaan [61] and msm [62] packages.

## 3. Results

Table 1 shows the demographic breakdown by child sex. Prenatal SES, SES at age 6, and mother’s IQ did not differ by child sex. Further, four out of the six WM indicators do not differ by sex. Females had a more inefficient CANTAB SWM strategy compared with males (*p* = 0.013). Females had shorter DMTS correct choice latency times compared to males (*p* = 0.022), with shorter latency times suggesting better performance on the task. In general, we see mobility between SES levels from pregnancy to age 6 (see Supplemental S1, which illustrates the transitions in SES level from prenatal to age 6), suggesting that current SES (at age 6) should be included as a covariate when assessing the association between prenatal SES and childhood WM.

The correlations between WM indicators are presented in Supplemental S2. The one-factor CFA provided a good fit to the data (chi-squared test *p* = 0.077; CFI = 0.995; RMSEA = 0.042; and SRMR = 0.017). All the factor loadings were statistically significant at the *p* = 0.05 level, and the standardized factor loadings ranged from −0.43 to 0.73. The tests of measurement invariance by sex supported strong invariance (see Supplemental S3, which shows the assessment of measurement invariance by sex), indicating that the means of the WM latent variable can be compared across sex.

**Figure 1:**
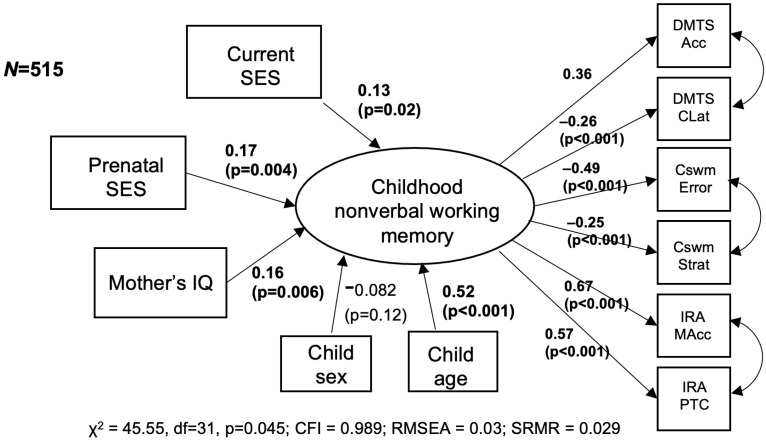
Associations of prenatal SES and childhood nonverbal WM, adjusted for maternal IQ, child sex, age, and current SES. Standardized factor loadings and *p*-values are presented.

Figure 1 presents the SEM to assess adjusted associations between prenatal SES and WM. The model fit indices were the following: chi-squared test *p*-value = 0.045; CFI = 0.989; RMSEA = 0.03; and SRMR = 0.029. A higher prenatal SES level was associated with higher WM (standardized factor loading = 0.17; *p* = 0.004), adjusted for mother’s IQ, child sex, child age, and current SES level. Further, a higher current SES was also associated with higher WM (standardized factor loading = 0.13; *p* = 0.02). Mother’s IQ and child age were also significant at the *p* = 0.05 level. As this model treats prenatal SES as an ordinal variable with six levels, we conducted a sensitivity analysis in which we considered an alternative coding for prenatal SES. We dichotomized prenatal SES (high—belonging in A/B, C+, C or D+ vs. low—belonging in D or E). The model fit indices were the following: chi-squared test *p*-value = 0.027; CFI = 0.99; RMSEA = 0.03; and SRMR = 0.03. In this adjusted model, high prenatal SES was still associated with higher WM (standardized factor loading = 0.13; *p* = 0.026). In separate adjusted regressions of each nonverbal working memory task measure (see Supplemental S4, which shows adjusted regressions of SES with each task variable separately), prenatal SES was associated with the CANTAB spatial working memory strategy, IRA memory accuracy, and IRA percent of task complete at the alpha = 0.05 significance level. After false discovery rate (FDR) correction, prenatal SES was only associated with IRA memory accuracy.

**Figure 2:**
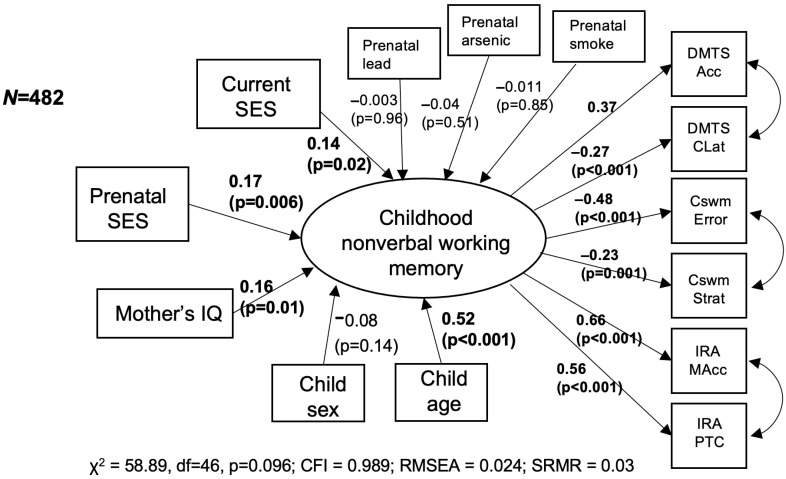
Associations of prenatal SES and childhood nonverbal WM, adjusted for maternal IQ, child sex, age, current SES, and prenatal exposure to neurotoxicants (lead, arsenic, secondhand smoke). Standardized factor loadings and *p*-values are presented.

Figure 2 presents the sensitivity analysis in which we additionally adjusted for prenatal exposure to neurotoxicants (maternal blood lead and arsenic levels, and self-reported exposure to secondhand smoke). The sample size was reduced to N = 482 due to missing values in the neurotoxicant exposure data. Higher prenatal SES was still associated with higher WM (standardized factor loading = 0.17; *p* = 0.006), as was higher current SES (standardized factor loading = 0.14; *p* = 0.02).

**Figure 3:**
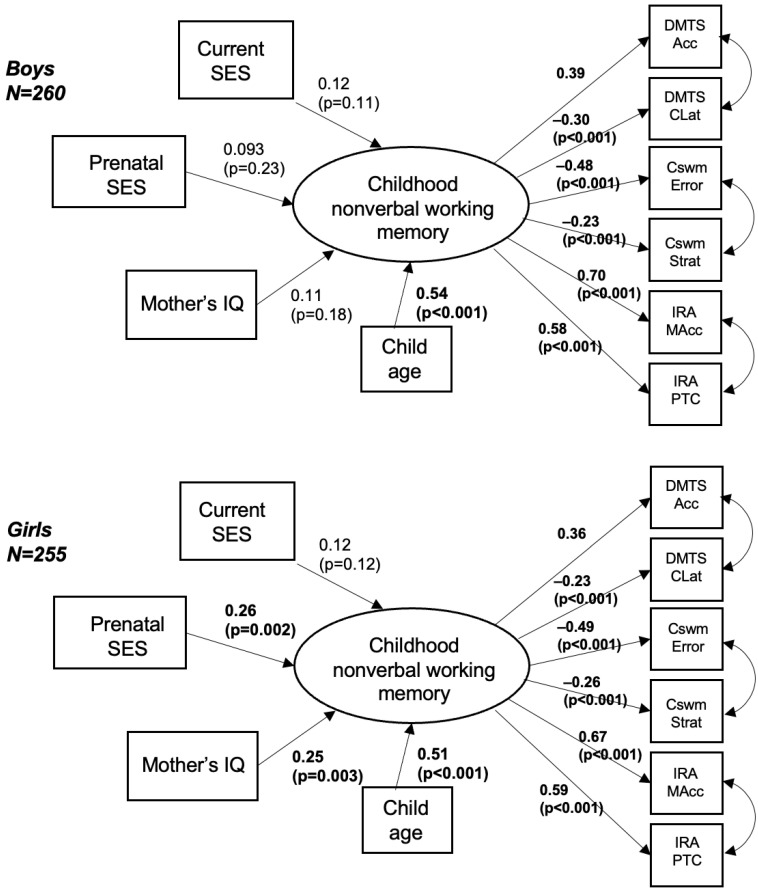
Sex-stratified associations of prenatal SES and childhood WM adjusted for maternal IQ, child sex, age, and current SES. Standardized factor loadings and *p*-values are presented.

Lastly, we analyzed whether the association between prenatal SES and WM is modified by child sex. For the main model, in which we adjusted for maternal IQ, child age, and current SES, there was indication of effect modification by sex (*p* = 0.052). As the previous literature supports the reporting of stratified analyses when the effect in one stratum is null [63], we present sex-stratified results in Figure 3. Among the N = 255 female participants, higher prenatal SES was significantly associated with higher WM (standardized factor loading = 0.26; *p* = 0.002), as well as child age with higher WM (standardized factor loading = 0.51, *p* < 0.001) and maternal IQ (standardized factor loading = 0.25, *p* = 0.003). Among the N = 260 male participants, only child age was a significant predictor of WM (standardized factor loading = 0.54, *p* < 0.001).

## 4. Discussion

In this analysis using a prospective cohort in Mexico, we assessed whether prenatal SES may be a predictor of childhood WM. We used multiple variables from three WM tasks to construct a latent variable of WM for increased measurement precision. Independent of current childhood SES levels, prenatal SES was a significant predictor for childhood WM. As SES is thought to be in part a proxy for environmental exposures, we conducted a sensitivity analysis by including prenatal exposure to multiple neurotoxicants. Our findings suggest that prenatal SES significantly predicts childhood WM even when adjusting for select prenatal neurotoxicant exposures. Lastly, we found a suggestion of effect modification by sex, with prenatal SES being a significant predictor of childhood WM in girls but not in boys.

By creating an integrative measure of WM that incorporated two indicators from each WM task, we were able to obtain more interpretable findings on the associations of prenatal SES with childhood WM. If we had assessed each task variable separately, prenatal SES was only significantly associated with one out of six task variables after adjusting for multiple comparisons, making this association difficult to interpret. Many studies have found significant positive association between socioeconomic status and childhood working memory [27,64,65], and such association does not vary by age [27,64]. Lower SES is found to be associated with poorer working memory [27,64], including worse working memory accuracy and slower reaction time [65]. While our study provides a prospective study of how SES impacts later childhood working memory, our findings may be limited by unmeasured confounding and loss-to-follow-up.

We found that for our cohort, SES was dynamic with substantial changes from pregnancy to childhood for some individuals; in general, there was upward mobility. By accounting for SES measured at different life stages, we were able identify prenatal effects that were independent of current SES levels.

Further research is needed to assess potential mechanisms by which prenatal SES may impact WM. Underlying stress may be a mechanism by which prenatal SES affects WM, with maternal stress shown to causally impact offspring WM in animal studies [66,67,68] with sex-dependent effects [66]. As there is sexual dimorphism [69] in brain development and neurodevelopment, we conducted an exploratory analysis to study whether there were sex-specific differences in how SES affects childhood WM. Our findings indicate that there may be a greater impact on girls compared with boys, and it may be of interest in future research to study sex-specific differences in replication datasets or larger cohorts.

Our analysis found that an integrative measure of working memory, combining multiple task variables, may provide more interpretable findings to study risk and protective factors of working memory. Psychometric approaches, such as structural equation modeling, may provide a robust modeling framework to identify novel risk and protective factors for child neurodevelopment.

## Figures and Tables

**Table 1:** Summary of variables by child sex.

	Boys	Girls	*p*-Value
Sample size (N)	260	255	
DMTS overall accuracy (median [IQR])	86.27 [81.82, 91.23]	87.50 [82.03, 91.75]	0.155
CANTAB SWM between search errors	63.00 [55.75, 71.00]	65.00 [58.00, 71.00]	0.449
CANTAB SWM strategy	39.00 [37.00, 41.00]	40.00 [38.00, 41.00]	0.013
DMTS overall correct choice latency time	3.08 [2.45, 4.12]	2.87 [2.34, 3.78]	0.022
IRA memory accuracy	72.32 [51.43, 82.10]	69.94 [47.09, 81.05]	0.118
IRA percent of task complete	100.00 [66.67, 100.00]	100.00 [66.67, 100.00]	0.119
Age (years)	6.56 [6.30, 7.07]	6.56 [6.29, 7.01]	0.761
Prenatal SES (%)			0.235
E (lowest)	27 (10.4)	23 (9.0)	
D	115 (44.2)	112 (43.9)	
D+	57 (21.9)	63 (24.7)	
C	36 (13.8)	34 (13.3)	
C+	25 (9.6)	18 (7.1)	
A/B (highest)	0 (0.0)	5 (2.0)	
Mother’s IQ (median [IQR])	86.00 [75.00, 95.00]	86.00 [77.50, 94.00]	0.751
SES at age 6 (%)			0.282
D− (lowest)	1 (0.4)	1 (0.4)	
D	47 (18.1)	56 (22.0)	
D+	93 (35.8)	67 (26.3)	
C−	58 (22.3)	67 (26.3)	
C	36 (13.8)	45 (17.6)	
C+	20 (7.7)	15 (5.9)	
A/B (highest)	5 (1.9)	4 (1.6)	

IQR = interquartile range; DMTS = delayed match to sample; IRA = incremental repeated acquisition; CANTAB = Cambridge Neuropsychological Test Automated Battery; SWM = spatial working memory; SES = socioeconomic status.

## Data Availability

Data and/or computing code will be provided upon reasonable request to the corresponding author due to privacy reasons.

## Supplementary Materials

The following supporting information can be downloaded at: https://www.mdpi.com/article/10.3390/children12050537/s1, S1: Table that describes transitions in SES level from prenatal to age 6; S2: Figure that describes correlations between WM indicators; S3: Table that shows assessment of measurement invariance by child sex for the WM latent variable; S4: Table that shows adjusted associations of prenatal SES with each task outcome separately.
