# Supplementary material for: Associations of Prenatal Socioeconomic Status and Childhood Working Memory: A Structural Equation Modeling Approach
Source: Children (Basel). 2025 Apr 23;12(5):537. doi: 10.3390/children12050537 (PMC12110185; doi:10.3390/children12050537)
Supplement: Supplementary file 1 [file children-12-00537-s001.zip › children-3488706-supplementary.pdf]

Supplementary Materials:

Associations of Prenatal Socioeconomic Status and Childhood Working Memory: A Structural Equation Modeling Approach

Shelley H. Liu, David Bellinger, Kristen Dams-O'Connor, Jeanne A. Teresi, Ivan Pantic, Sandra Martínez-Medina, John Chelonis, Martha M. Téllez-Rojo and Robert O. Wright

Table of Contents:

- Supplemental S1. Table that describes transitions in SES level from prenatal to age 6
- Supplemental S2. Figure that describes correlations between WM indicators
- Supplemental S3. Table that shows assessment of measurement invariance by child sex for the WM latent variable
- Supplemental S4. Table that shows adjusted associations of prenatal SES with each task outcome separately

Supplemental S1: Transitions in SES level from prenatal to age 6. At pregnancy, SES is measured by a six category index, developed by the Mexican Association of Marketing Research and Public Opinion Agencies (AMAI). At 6 yrs, SES is measured by an updated 7-category AMAI index. Based on N=515. The table presents the number of participants who transitioned from one category during pregnancy, to another category at 6 years.

For example, for participants in the lowest SES level during pregnancy (level E), only 1 stayed in the lowest level at 6 years (D-). 18 transitioned to the next highest level (D), 19 transitioned to D+, 6 transitioned to C-, 5 transitioned to C, and 1 transitioned to C+.

		**SES at 6 Yrs (7-category AMAI)**						
		**To**						
**Prenatal SES (6-cat AMAI)**	**From**	*D- (lowest)*	*D*	*D+*	*C-*	*C*	*C+*	*A/B (highest)*
	E (lowest)	1	18	19	6	5	1	0
	D	1	43	69	63	35	13	3
	D+	0	23	40	32	19	4	2
	C	0	14	15	14	12	13	2
	C+	0	5	14	10	10	2	2
	A/B (highest)	0	0	3	0	0	2	0

Supplemental S2: Correlations between WM indicators.

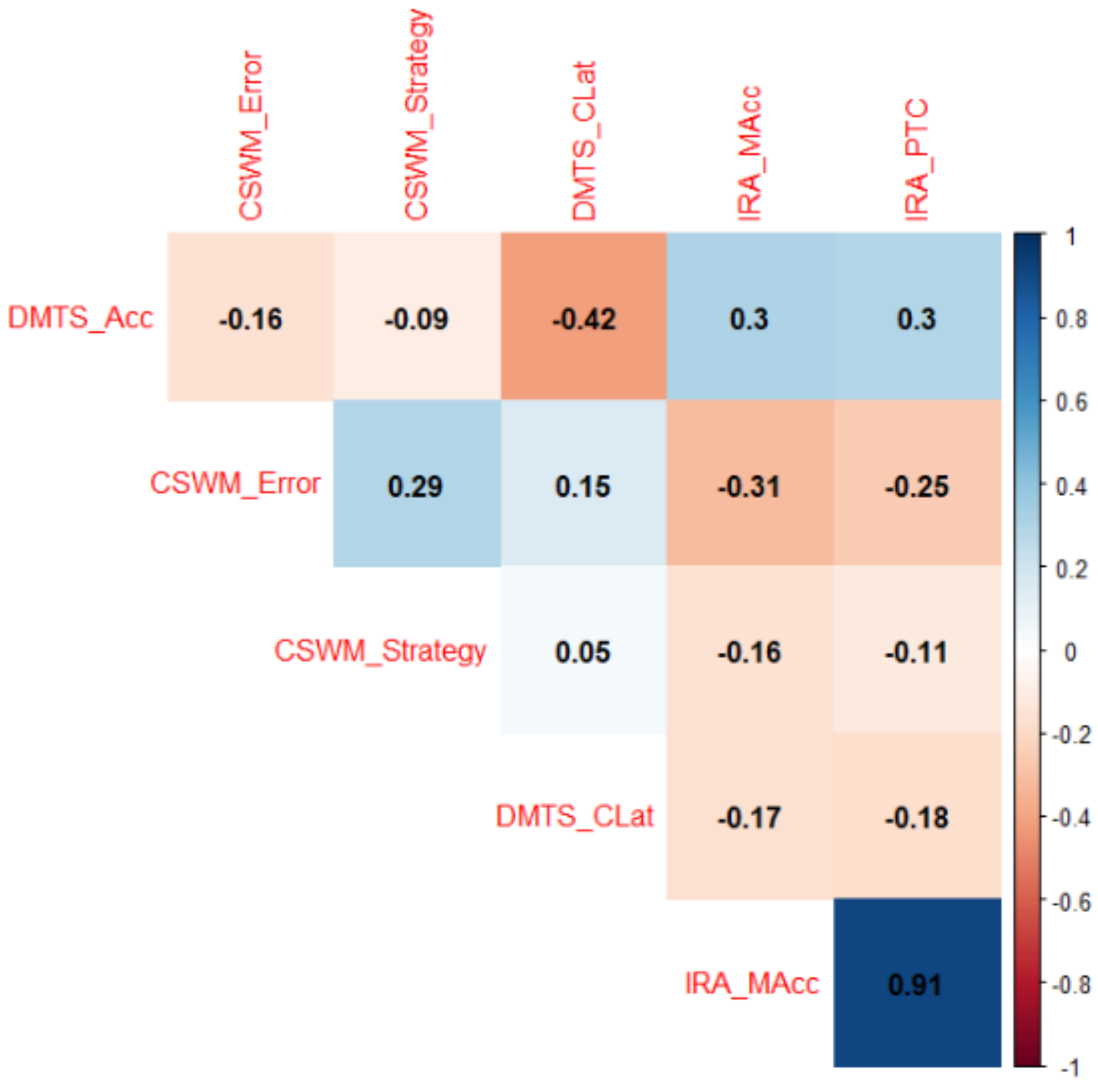

Supplemental S3: Assessment of measurement invariance by child sex for the WM latent variable. None are significant. This supports strong invariance (factor loadings and intercepts are the same across sex).

	Df	AIC	BIC	Chisq	Chisq diff	Df diff	Pr(>Chisq)
Configural Invariance	12	21367.196	21545.45	15.81183	NA	NA	NA
Weak Invariance	17	21361.255	21518.29	19.87088	4.059048	5	0.540946
Strong Invariance	22	21360.082	21495.9	28.69713	8.826254	5	0.116198

Predictors	CANTAB SWM Error *Estimates*	*p*	CANTAB SWM Strategy *Estimates*	*p*	DMTS Accuracy *Incidence Rate Ratios*	*p*	DMTS Correct Choice Latency *Incidence Rate Ratios*	*p*	IRA Memory Accuracy *Incidence Rate Ratios*	*p*	IRA Percent Task Complete *Incidence Rate Ratios*	*p*
(Intercept)	115.54	**<0.001**	43.52	**<0.001**	539.35	**<0.001**	154.87	**<0.001**	13.89	**<0.001**	25.25	**<0.001**
Prenatal SES	-0.84	0.079	-0.27	**0.03**	1	0.991	0.99	0.671	1.04	**0.006**	1.03	**0.027**
Child Sex												
Males	Ref											
Females	0.3	0.776	0.67	**0.013**	1.01	0.667	0.96	0.291	0.95	0.09	0.96	0.244
Maternal IQ	-0.11	**0.015**	-0.01	0.657	1	0.103	1	0.41	1	0.101	1	0.462
Current SES	-0.88	**0.038**	0.06	0.614	1.01	0.073	0.98	0.285	1.02	0.114	1.02	0.094
Child Age (years)	-5.67	**<0.001**	-0.67	**0.004**	1.04	**0.02**	0.83	**<0.001**	1.21	**<0.001**	1.16	**<0.001**
Observations	515		515		515		515		515		515	

Supplemental S4. In separate adjusted regressions of each nonverbal working memory task outcome variable, prenatal SES was associated with CANTAB spatial working memory strategy, IRA memory accuracy, and IRA percent task complete. After false discovery rate (FDR) correction, prenatal SES was only associated with IRA memory accuracy. We used linear regression for CANTAB measures, which were normally distributed. Due to skewness, we first log-transformed the other measures. However, the skewness was not mitigated by using log-transform, so we used negative binomial regression which accounts for skewed count variables. In order to make ours count variables, we multiplied each of the remaining variables by 10, and then rounded to the nearest integer.
